# Food addiction in Bulimia Nervosa: Analysis of body composition, psychological and problematic foods profile

**DOI:** 10.3389/fpsyt.2022.1032150

**Published:** 2022-10-20

**Authors:** Lucero Munguía, Lucía Camacho-Barcia, Anahí Gaspar-Pérez, Roser Granero, Carla Galiana, Susana Jiménez-Murcia, Carlos Dieguez, Ashley Nicole Gearhardt, Fernando Fernández-Aranda

**Affiliations:** ^1^Department of Psychiatry, University Hospital of Bellvitge, Barcelona, Spain; ^2^Psychoneurobiology of Eating and Addictive Behaviors Group, Neurosciences Program, Bellvitge Biomedical Research Institute (IDIBELL), Barcelona, Spain; ^3^CIBER Fisiopatología Obesidad y Nutrición (CIBERobn), Instituto de Salud Carlos III, Barcelona, Spain; ^4^Department of Psychobiology and Methodology, Autonomous University of Barcelona, Barcelona, Spain; ^5^Department of Clinical Sciences, School of Medicine and Health Sciences, University of Barcelona, Barcelona, Spain; ^6^Department of Physiology, Centro Singular de Investigación en Medicina Molecular y Enfermedades Crónicas (CIMUS), Instituto de Investigación Sanitaria, University of Santiago de Compostela, Santiago de Compostela, Spain; ^7^Department of Psychology, University of Michigan, Ann Arbor, MI, United States

**Keywords:** food addiction, Bulimia Nervosa, emotion regulation, problematic foods, body composition

## Abstract

**Introduction:**

Food Addiction (FA) has been related with eating disorders (ED), especially Bulimia Nervosa (BN). BN + FA may have different physical characteristics than patients with BN without the comorbidity, such as body mass index (BMI) or body composition, and psychological as emotion regulation. However, the relationship between psychological and physical aspects, connected by problematic food and its influence on body composition, has been barely studied. Therefore, the aims of the present study are:

**Aims:**

(a) To explore the differences in body composition between FA positive (FA+) and negative (FA–) in women with BN; (b) to identify problematic relationship with certain food types, according with the foods mentioned in the YFAS scale questionnaire, between FA+ and FA– patients; (c) to know the psychological characteristic differences between FA+ and FA– patients, considering emotion regulation, personality traits and general psychopathological state; (d) to identify the relationship between physical and psychological traits, and the identified problematic foods, in patients with BN and FA.

**Methodology:**

*N* = 81 BN women patients, with a mean age of 29.73 years ± 9.80 SD, who completed the questionnaires: Yale Food Addiction Scale V 1.0 (YFAS 1.0), Eating Disorder Inventory-2 (EDI-2), Symptom Checklist-90 Items-Revised (SCL-90-R), and Difficulties in Emotion Regulation Strategies (DERS). YFAS problematic foods were grouped considering their principal nutrients sources. Body composition and difference in metabolic age was determined using bioimpedance analyzer.

**Results:**

The 88% of patients with BN presented FA+. Patients with BN who were FA+ self-reported more problematic relationships with sweets and starches. Also presented higher emotion regulation difficulties, general psychopathology and eating symptomatology severity, than those without FA. Finally, emotional regulation difficulties were positively associated with higher eating disorder symptomatology and more types of foods self-reported as problematic, which increased indirectly fat mass.

**Conclusion:**

The results suggest that BN + FA presented more eating and psychopathology symptomatology and higher problems with specific food types. As well, the path analysis emphasized that emotion regulation difficulties might be related with problematic food relationship in BN, impacting over the ED severity.

**Implications:**

The results may impact the development of precise therapies for patients with BN + FA.

## Introduction

Food addiction (FA) has been described as the presence of maladaptive eating behaviors consistent with addictive processes, mainly characterized by excessive consumption of ultra-processed foods. While several studies have reported FA in non-clinical samples (general adult population and student samples), the prevalence of FA is higher in populations with obesity, and in Eating Disorders (ED) patients, especially the ones in the binge spectrum disorders, as Bulimia Nervosa (BN) (86–96%) (1–3).

Frequently, in BN higher FA scores have been positively associated with greater ED severity (4, 5), more general psychopathology (5, 6) and greater dysfunctional personality traits (6, 7). Furthermore, Emotion Regulation (ER) difficulties and greater impulsivity seem to be an important characteristic in this population with absence of FA (FA–). Negative urgency has been found in the presence of FA (FA+) and binge spectrum disorders patients (8, 9). Negative urgency represents the emotional-related aspect of impulsivity, indicating the tendency to act rashly and engage in problematic behavior (in the case of FA, the disorder eating patterns, and excessive food intake) as a response to a negative emotional state (10).

It is important to mention that recent studies have identified different subgroups of patients with ED and FA. Considering a cross-sectional and a longitudinal approach, Cluster-based analyses have found that FA is most commonly associated with BN (relative to other types of disordered eating) (7, 11), which predicts poorer treatment response, as higher rates of dropouts (11). These results are consistent with other studies that have found that patients with BN + FA had worse prognosis than patients with BN without FA (2).

In BN, abnormal eating patterns and excessive compensatory behaviors may result in alterations in body composition. It has been previously reported that patients with bulimia had significantly higher total daily energy intake compared with control subjects (12). Further, certain groups of women with BN consumed large meals primarily of desserts and snack foods (13), which may contribute to higher BMI and greater accumulation of adipose tissue. However, other study on body composition has found that normal weight patients with BN display no significant differences in body composition when compared to healthy normal weight controls (14).

Regarding the influence of FA on body composition, the evidence suggests that individuals with FA and obesity present higher weight and had greater total body fat and trunk fat than non-FA individuals (15), however, it has not been previously reported whether the presence of FA may play a role in the body composition of patients with BN. Even though people with BN might present a normal weight in the majority of cases (16), the presence of comorbid FA could be affecting total body fat percentage. Previous studies have associated the excess accumulation of body fat in normal weight individuals with adverse metabolic profiles, including dyslipidemias and cardiometabolic dysregulations (17–19). A more comprehensive clinical profile of BN + FA patients, including psychological, nutritional and body composition features, might be needed in order to establish a more personalized treatment approach for this group.

In order to explore this, is important to consider certain foods as potentially addictive. Regarding FA model, this matter is still under debate. Studies of non-ED patients have found that ultra-processed foods, high in refined carbohydrates and/or added fats are typically endorsed as the most addictive, but investigations into this topic in ED samples are limited (20, 21).

Therefore, the present study has as aims: (a) to explore the differences in body composition between BN + FA and BN-FA patients; (b) to identify problematic food relationships, according to the foods identified as most addictive on the YFAS, between BN + FA and BN-FA; (c) to determine the clinical differences in both groups, considering emotion regulation, personality traits and general psychopathological state; (d) to identify the relationship between physical and psychological traits, and the considered problematic foods, in BN with FA patients, in order to determine a complete clinical profile in this population.

We hypothesize that those BN + FA patients, when compared to BN-FA, will present higher BMI and higher total body fat percentage, as well as worst general psychopathological state, higher severity of the disorder, and more emotional regulation difficulties. We also expect that the food referred with a problematic relationship will be different between BN + FA and BN-FA, where those patients with comorbid FA will report higher problematic associations to high fats and refined carbohydrates food groups. Finally, that FA will not only be related with the psychological variables, contributing to a general worst profile, as mentioned above, but will also have more problematic food relationships, as well as differences in the body composition.

## Materials and methods

### Sample and procedure

The present cross-sectional study was conducted in a sample composed of female outpatients diagnosed with BN (*n* = 81), with a mean age of 29.22 years ± 9.80 SD. Participants were recruited from the Eating Disorders Unit of the University Hospital of Bellvitge – Barcelona, who attended to request treatment or were already linked to it, from 2013 to 2016. Diagnosis of BN was made by senior clinicians through a DSM-5 diagnostic criteria semi-structured clinical interview (SCID-5) (22). Participants were evaluated trough a psychometric battery that included different questionnaires (see section “Psychometric measures”). All evaluations were performed by experienced psychologists and psychiatrists.

In accordance with the Declaration of Helsinki, the present study was approved by the Clinical Research Ethics Committee of the University Hospital of Bellvitge. During the first evaluation session, participants are informed that several research studies are carried out in the ED unit, and thought informed consent, they accepted the data of their evaluations to be used with clinical and research purpose. Only participants that singed an informed consent were included in the study.

### Assessment

#### Psychometric measures

*Yale Food Addiction Scale V 1.0 (YFAS 1.0)*, (23)*;* validated in Spanish population (5). The YFAS questionnaire was validated for a clinical population in 2009 and was subsequently validated in Spanish. YFAS is based on the criteria of the Diagnostic and Statistical Manual of Mental Disorders, that measures levels of substance dependence, where the term “substances” is replaced by “certain food” and is used for the quantification of the severity of FA during the previous 12 months. This questionnaire assesses seven diagnostic criteria for dependence in case of excessive consumption of high-fat and/or high-sugar foods. It uses two scoring systems: a count of FA symptoms (from 0 to 7, according to DSM-IV-TR diagnostic criteria) and for the assessment of the diagnosis of FA (3 or more symptoms plus clinically significant impairment/distress). The measures consisted of 25 items answered on an 8-point Likert scale. The internal consistency of the total scale for our sample was 0.820 (coefficient alpha).

*Eating Disorders Inventory 2 (EDI-2)* (24). Is reliable and valid self-report questionnaire that assesses different cognitive and behavioral characteristics typical of ED in eleven subscales: drive for thinness, bulimia, body dissatisfaction, inefficacy, perfectionism, interpersonal distrust, interoceptive awareness, fear of maturity, asceticism, impulse regulation, and social insecurity. The measures consist of 91 items, answered on a 6-point Likert scale. The internal consistency of the total scale for our sample was 0.955 (coefficient alpha).

*Symptom Checklist-90-Revised (SCL-90-R)* (25). The SCL-90-R questionnaire is used to assess a wide range of psychological problems and symptoms of psychopathology considering nine primary symptom dimensions: somatization, obsessive-compulsive, interpersonal sensitivity, depression, anxiety, hostility, phobic anxiety, paranoid ideation, and psychoticism; and includes three global indices: global severity index (general psychological distress), positive symptom distress index (the intensity of symptoms), and a positive symptom total (self-reported symptoms). The global severity index was used as a test summary. The measure consists of 90 items answered on a 5-point Likert scale. The internal consistency of the subscales for our sample was 0.969 (coefficient alpha).

*Difficulties in Emotion Regulation Scale (DERS)* (26), validated in Spanish population (27). The DERS questionnaire assesses emotion dysregulation in six subscales: non-acceptance of emotional responses, difficulties in pursuing goals when experiencing strong emotions, difficulties in controlling impulsive behaviors when experiencing negative emotions, lack of emotional awareness, limited access to emotional regulation strategies and lack of emotional clarity. The measure consists of 36 items and is answered on a 5-point Likert scale. The internal consistency of the subscales for our sample ranges between 0.796 and 0.924, and the one for the total score was 0.950 (coefficient alpha).

#### Anthropometric parameters and body composition

Both height and weight were measured by trained personnel and used to determine the body mass index (BMI), calculated as weight (Kg)/[height(m)]^2^. Body composition was determined using the bioimpedance analysis, a practical, inexpensive, and non-invasive method, utilizing a Multifrequency TANITA MC-80 body impedance device (TANITA, Japan). Values of total fat, non-fat, muscle and bone mass, as body water percentage was analyzed. Basal metabolic rate and metabolic age (Met-age) were determined by the device. Met-age was used to calculate difference in age (participants’ chronological age *minus* participants Met-age). Metabolic age has been previously proposed as an indicator of inflammation and cardiovascular risk (28).

#### Problematic food groups

To identify the problematic relationships generated by certain food groups, the last item of the YFAS questionnaire was considered. This item presents a list of different foods so that the participants can indicate those that him/her recognizes as problematic. These foods were then grouped into five categories according to the possible addicting nutritional content of their chemical composition: high in fat and simple sugars, high in fat and sodium, high in starch, high in simple sugars, and control foods (low in fat, sodium, starch, and simple sugars) (Table 1).

**TABLE 1:** Distribution of the problematic foods included in YFAS.

Food groups	Foods included in YFAS
High fat/High sugars	ice cream, chocolate, cookies, cake, donuts
High fat/High sodium	bacon, burgers, pizza, French fries, steak, cheeseburgers
High starches	white bread, pasta, rice, rolls
High in sugars	candies, soft drinks
Controls	apples, broccoli, lettuce, strawberries, carrots, bananas

### Statistical analysis

Statistical analysis was done with Stata17 for Windows (29). The comparison between the groups defined for the screening score in the YFAS (FA− versus FA+) was done with chi-square (χ^2^) tests for categorical measures and *t*-tests for quantitative measures (exact p-values were obtained for proportion comparisons with expected counts lower than 5 and for mean comparisons with non-normal distribution). In addition, these analyses included the calculation of the effect size through the standardized Cohen coefficient (*h*-value for proportion comparisons and *d*-value for mean comparisons, considering the thresholds 0.50 and 0.80 for moderate and large effect size) (30). The association between the FA severity level (YFAS total score) with body composition and other clinical measures was estimated with the correlation coefficients. Due the solid association between the significance level for the *R*-coefficients and the sample size, effect size was considered low-poor | *R*| > 0.10, moderate-medium for | *R*| > 0.24 and large-high for | *R*| > 0.37 (31).

Path analysis was implemented through Structural Equation Modeling (SEM). All the parameters were free-estimated, and a latent variable measuring the problematic foods profile was defined based on the different foods analyzed in this work. With the aim to obtain a final parsimonious model (with the highest statistical power), parameters with not significant tests were deleted and the model was re-specified and re-adjusted (only non-significant coefficients were retained for the measurement parameters related to the latent variable). Goodness-of-fit was evaluated using standard statistical measures, and it was considered adequate for (32): non-significant result in the χ^2^ test, root mean square error of approximation RMSEA < 0.08, Bentler’s Comparative Fit Index CFI > 0.90, Tucker-Lewis Index TLI > 0.90, and standardized root mean square residual SRMR < 0.10The global predictive capacity of the model was measured by the coefficient of determination (CD).

## Results

### Association of food addiction with problematic food groups, body composition and clinical profile

The first block of Table 2 contains the distribution of all the variables of the study among the total sample. The mean for BMI was 24.2 kg/m2 (SD = 4.9). The problematic relationship with high fat/high sugars food was reported by 93.8% of the patients, high fat/high sodium foods by 88.9%, high starches by 82.7% and high sugars by 61.7%. Control foods were endorsed by 22.2%.

**TABLE 2:** Association between food addiction (FA) screening scores and clinical profile.

	Total *(n* = *81)*		FA − *(n* = *9)*		FA + *(n* = *72)*			
	*Mean*	*SD*	*Mean*	*SD*	*Mean*	*SD*	*p*	*|d|*
Fat mass	26.88	7.60	25.03	4.08	27.11	7.92	0.441	0.33
Non-fat mas	44.64	3.65	44.50	2.30	44.66	3.80	0.901	0.05
Muscle mass	42.62	3.50	42.45	2.25	42.64	3.64	0.880	0.06
Body water%	51.13	4.79	52.48	2.61	50.96	4.99	0.372	0.38
Bone mass	2.27	0.18	2.27	0.12	2.27	0.19	0.993	0.00
Basal metabolic rate	1369	123.0	1370	61.0	1369	128.9	0.975	0.01
Metabolic age	27.16	12.86	21.04	7.60	27.93	13.20	0.130	**0.64^†^**
Difference in age	2.06	10.18	1.52	6.22	2.13	10.60	0.867	0.07
BMI (kg/m^2^)	24.18	4.88	23.16	1.63	24.30	5.13	0.513	0.30
EDI Drive thinness	15.21	4.51	11.56	4.45	15.67	4.33	0.009*	**0.94^†^**
EDI Body dissatisfaction	17.27	7.38	14.56	6.35	17.61	7.47	0.244	0.44
EDI Interoceptive awareness	12.69	6.25	7.78	5.72	13.31	6.07	0.011*	**0.94^†^**
EDI Bulimia	10.36	4.35	6.44	3.57	10.85	4.20	0.004*	**1.13^†^**
EDI Interpersonal distrust	5.60	4.52	2.67	2.40	5.97	4.60	0.038*	**0.90^†^**
EDI Ineffectiveness	12.06	6.69	9.56	4.98	12.38	6.84	0.236	0.47
EDI Maturity fears	7.94	4.59	8.00	2.92	7.93	4.78	0.966	0.02
EDI Perfectionism	6.38	4.56	3.78	2.28	6.71	4.68	0.069	**0.80^†^**
EDI Impulse reg.	6.79	5.17	4.78	5.65	7.04	5.10	0.218	0.42
EDI Ascetic	7.94	4.04	5.00	2.60	8.31	4.05	0.020*	**0.97^†^**
EDI Social insecurity	8.10	5.09	5.67	2.96	8.40	5.23	0.129	**0.64^†^**
EDI Total	110.35	39.14	79.78	28.00	114.17	38.79	0.012*	**1.02^†^**
SCL-90R Somatic	1.83	0.76	1.29	0.92	1.89	0.72	0.023*	**0.73^†^**
SCL-90R Obs.-com	1.97	0.72	1.44	1.01	2.04	0.66	0.017*	**0.71^†^**
SCL-90R Sensitivity	2.16	0.69	1.68	0.75	2.22	0.66	0.026*	**0.76^†^**
SCL-90R Depress.	2.40	0.74	1.84	0.91	2.47	0.69	0.016*	**0.77^†^**
SCL-90R Anxiety	1.62	0.69	1.16	0.83	1.67	0.66	0.034*	**0.69^†^**
SCL-90R Hostility	1.22	0.78	0.72	0.52	1.28	0.79	0.041*	**0.84^†^**
SCL-90R Phobic	0.97	0.66	0.80	0.72	0.99	0.66	0.433	0.27
SCL-90R Paranoia	1.45	0.72	1.23	0.92	1.48	0.69	0.328	0.31
SCL-90R Psychotic	1.34	0.55	1.20	0.68	1.36	0.54	0.408	0.27
SCL-90R GSI	1.79	0.56	1.37	0.78	1.84	0.52	0.017*	**0.72^†^**
SCL-90R PST	65.16	12.73	57.78	21.12	66.08	11.16	0.065	**0.51^†^**
SCL-90R PSDI	2.41	0.50	1.97	0.56	2.46	0.47	0.005*	**0.94^†^**
DERS Non-acceptance	19.83	6.06	17.44	4.80	20.13	6.16	0.213	**0.51^†^**
DERS Directed goals	17.30	4.69	16.67	4.06	17.38	4.78	0.672	0.16
DERS Impulse	18.15	4.81	15.56	2.96	18.47	4.92	0.087	**0.72^†^**
DERS Awareness	17.23	4.05	18.44	2.01	17.08	4.22	0.345	0.41
DERS Strategy	26.37	6.65	23.44	5.22	26.74	6.74	0.163	**0.55^†^**
DERS Lack of clarity	14.60	4.34	13.44	3.71	14.75	4.41	0.398	0.32
DERS Total	113.48	23.40	105.00	17.33	114.54	23.94	0.251	0.46

*Bold: significant comparison. ^†^Bold: effect size into the mild to large range.

**FIGURE 1:**
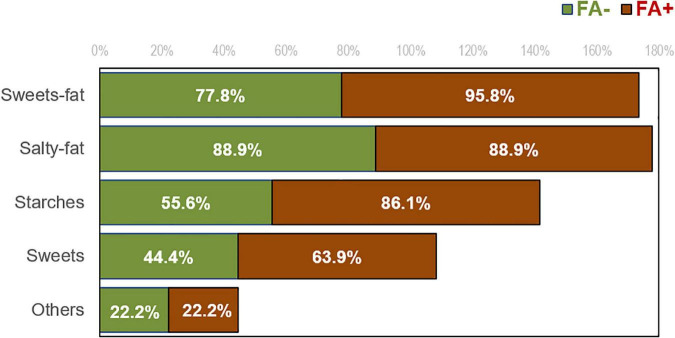
Distribution of the self-reported problematic type of foods in patients with FA negative and positive score.

Figure 1 displays the bar-charts with the distribution of the problematic food groups, for patients with and without FA, according to the YFAS screening tool. Overall, patients with a positive FA score increase the likelihood of recognize as problematic all types of food, although only significant differences were obtained for sweets with high fat content (*p* = 0.034, *| h|* = 0.57) and starches (*p* = 0.022, *| h|* = 0.70). However, the control foods were endorsed the least of all food groups and there were no differences between the FA+ and FA– groups (22.2% of individuals in both groups endorsed a problem with a control food).

Table 2 contains the comparison of the body composition and clinical profile for women with BN and with FA+ versus FA–. Due the low sample size for the FA− group (11.25% of the sample), relevant differences were considered for significant differences (*p* < 0.05) or effect size within the ranges moderate to large (| *d|* > 0.50). No significant differences were observed in the body composition profile between the FA+ and FA– groups. Women within the FA+ group had a higher mean metabolic age, though no significant differences were observed in the difference with chronological age. The FA+ group also reported higher ED severity levels (EDI-2 drive for thinness, interoceptive awareness, bulimia, interpersonal distrust, perfectionism, ascetic, social insecurity and total scales), worse psychology state (higher means in all the SCL-90R scales, except for phobic anxiety, paranoid ideation and psychotic ideation), and worse performance in the emotion regulation (higher means in the DERS non-acceptance of emotional responses, impulse control difficulties, and limited access to emotion regulation strategies).

Table 3 includes the correlation matrix to assess the relationship between the YFAS-total score (as a dimensional measure for the FA severity level) with body composition and clinical profile. The YFAS-total was positively related with the total fat mass, metabolic age, the ED symptom severity levels as measured with the EDI-2 (except for ineffectiveness, maturity fears, perfectionism, impulse regulation, and social insecurity), the psychological distress as measured by the SCL-90R (sensitivity, depression, and the global indexes), and the difficulties in the emotional regulation (controlling impulsive behaviors when experiencing negative emotions and limit access to emotional regulation strategies). A negative correlation was also found between the YFAS-total and the percentage of body water.

**TABLE 3:** Correlation matrix.

	YFAS total		YFAS total
Fat mass	**0.289^†^**	SCL-90R Somatic	0.223
Non-fat mas	0.135	SCL-90R Obsessive-compulsive	0.217
Muscle mass	0.121	SCL-90R Sensitivity	**0.365^†^**
Body water%	**−0.299^†^**	SCL-90R Depression	**0.338^†^**
Bone mass	0.126	SCL-90R Anxiety	0.205
Basal metabolic rate	0.158	SCL-90R Hostility	0.215
Metabolic age	**0.304^†^**	SCL-90R Phobic	0.136
Difference in age	**−**0.107	SCL-90R Paranoia	0.096
BMI (kg/m^2^)	0.226	SCL-90R Psychotic	0.134
EDI Drive thinness	**0.357^†^**	SCL-90R GSI	**0.289^†^**
EDI Body dissatisfaction	**0.261^†^**	SCL-90R PST	**0.258^†^**
EDI Interoceptive awareness	**0.323^†^**	SCL-90R PSDI	**0.292^†^**
EDI Bulimia	**0.377^†^**	DERS Non-acceptance	0.204
EDI Interpersonal distrust	**0.274^†^**	DERS Directed goals	0.171
EDI Ineffectiveness	0.135	DERS Impulse	**0.246^†^**
EDI Maturity fears	0.120	DERS Awareness	**−** 0.056
EDI Perfectionism	**−**0.030	DERS Strategy	**0.244^†^**
EDI Impulse reg.	0.086	DERS Lack of clarity	0.115
EDI Ascetic	**0.312^†^**	DERS Total	0.219
EDI Social insecurity	0.189		
EDI Total	**0.317^†^**		

^†^Bold: effect size into the mild to large range.

### Path analysis

**FIGURE 2:**
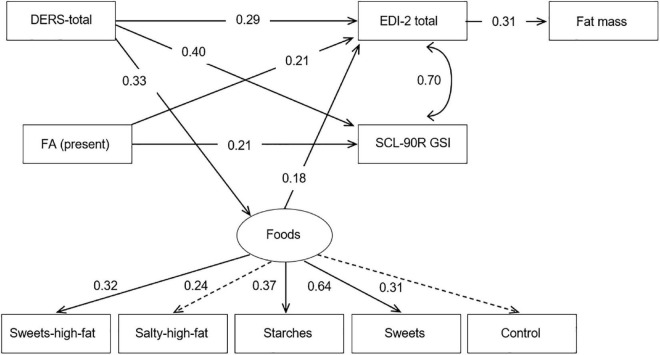
Standardized coefficients in the path-analysis. FA: food addiction (0 = absent; 1 = present). Fit statistics: χ^2^ = 28.98 (*p* = 0.413); RMSEA = 0.021 (95%CI: 0.001 to 0.080); CFI = 0.993; TLI = 0.989; SRMR = 0.080; CD = 0.306.

Figure 2 shows the path-diagram with the standardized coefficients. Adequate goodness-of-fit was achieved [χ^2^ = 28.98 (*p* = 0.413), *RMSEA* = 0.021 (95% confidence interval: 0.001 to 0.080), *CFI* = 0.993, *TLI* = 0.989, and *SRMR* = 0.080]. The global predictive capacity was around 31% (*CD* = 0.306). Supplementary Table 1 (Supplementary material) contains the complete results for the SEM (tests for the total, direct and indirect effects).

In this study, the latent variable measuring the foods profile was significantly defined by the observed variables sweets high fat, starches, and sweets (the coefficients for salty high fat and control foods obtained *p* < 0.05 values). Patients with more difficulties in the emotion dysregulation functions obtained higher ED severity levels, worse psychopathology state, and higher scores in the latent variable measuring the foods consumption profile. Women with FA + screening score also greater ED severity level and higher psychological distress. The higher fat mass levels were directly predicted by higher scores in the EDI-2 total. The ED severity level was also implied in some indirect relationships: (a) higher scores in the DERS total and being in the FA+ group impacted in the EDI-2 total score, which next contributed to the increase in the fat mass; and (b) higher scores in the latent variable measuring the foods consumption profile were associated with increased levels in the EDI-2 total, which next contributed on the fat mass.

## Discussion

The aims of the present study were to explore differences in body composition and psychological characteristics between BN with and without FA, whether there were differences in the self-reported problematic relationship with the different food types evaluated with the YFAS; and finally, in BN + FA patients, to identify the relationship between physical and psychological traits, and the food type considered problematic.

The majority of patients with BN endorsed FA (88.75%), which highlights the importance of investigating addictive mechanisms in this population (1, 6, 7). Despite the relatively small number of patients with BN who did not endorse FA, differences between those with and without FA still emerged.

Our results, regarding the association of FA with specific problematic food groups, showed that those patients with BN and FA+ significantly recognize high sugars foods, high fat content foods and starches as problematic. These findings are consistent with data obtained in a study that aimed to explored the differences in dietary preferences, through the dietary intake, among an adult population with and without FA (33). Their results regarding dietary behavior showed that a significantly higher proportion of people with FA+ reported higher intakes of high fat foods, such as snacks, fast food and chips, as well as greater intake of soft drinks, rich in sugars (33). This is also consistent with studies in non-clinical samples that find that foods with high levels of refined carbohydrates and/or added fats are indicated as the most addictive (20, 21). This is relevant to the current debate about whether the attributes of the food are an important factor in triggering addictive processes or whether the act of eating (regardless of food type) is more important (34). The current findings are consistent with the hypothesis that certain foods are more likely to be implicated in addictive patterns of intake, specifically processed foods with high levels of reinforcing ingredients (e.g., sugar, starches, fat).

Regarding the psychological characteristics, the present results were coincident with the literature in reference to a worst psychopathological general state, and, specially, more difficulties in ER, in BN + FA, when compared to BN-FA [T. (7–9, 11, 35)]. As well, the FA + groups presented higher ED severity; current literature have reported that FA was associated with more craving and binge episodes in BN (36). According to the FA model, this could be related with the type of foods that are consumed. In both, animals and human based studies, the consumption of high palatable foods and with a high glycemic load generate changes in the hunger and reward circuits (37–39). Current reviews have gone deeper into the study of how the addictive process can be activated by certain types of food in similar ways as in other addictive substances. They concluded that high palatable food could induced changes in the hedonic system resulting in craving for the substance (positive reinforcement), while tolerance is developed, and the desire of further activation increases as well, in order to avoid the negative effect of the non-consumption of the substance (negative reinforcement) (40).

Considering body composition, although BN + FA showed quasi-significant greater levels of fat mass and mean BMI, contrary to our hypothesis, those results were not statistically significant, when compared with BN-FA. Even though we expected to find dissimilarities when compared the FA+ and FA– groups, these results are consistent with that of Probst et al. (14) who concluded that BN women patients did not display differences in the body composition profile when compared to healthy controls (14). These may be due to our study small sample size, and further studies with a bigger cohort are needed to confirm them.

Finally, the path analysis that explores the associations of FA, psychological traits, and fat mass, show interesting results. The presence of FA, and the higher scores in the DERS total, both had an impact in the EDI-2 total score, which then contributed indirectly to the increased fat mass. This result could support the multicausality and multifunctionality of eating disorders, and the justification of biopsychosocial explanatory models, as has been suggested in previous studies (41). As well, these results open an important research line. We found that ER state was possible associated with the type of food considered problematic, which may be an introductory aspect to determine a different BN + FA profile, highly influenced by ER difficulties, due to only indirect results have been explored in this field (2).

Previous studies have found that the mood influences in the body idealization and dissatisfaction, besides the presence of high caloric food cues (42), and that the negative emotions triggered the desire to eat and the selection of high caloric foods (43, 44). Other authors have found, in laboratory controlled situations, that patients with FA increases their attention to food images after sad mood was induced (45). Therefore, future studies, from a broader neurobiological perspective, may analyze the underlying interaction of abnormal eating patterns and emotional regulation difficulties (46, 47) in BN, in order to confirm whether BN + FA patients, who have higher ER difficulties, may be a different BN subtype. In addition, measurement of specific neurobiological biomarkers could improve the identification of specific subset of patients.

### Limitations and future research lines

This study has a few limitations. Firstly, due to the cross-sectional design of the study causality cannot be conferred. Secondly, our cohort consisted of young women adults with BN, further studies would be required focusing on both sexes and wider age range including postmenopausal women and, finally, the low sample size of the BN-FA group that may compromise the generalization of the results.

However, an interesting research line could be open based on the current results. To our knowledge, there are few studies exploring the relationship between BN, FA, and body composition parameters that suggest the important relevance of ER processes. Empirically supported treatments for addiction commonly focus on helping individuals develop ER strategies (48), therefore, investigating whether targeted interventions for ER could also improve treatment outcomes for BN + FA individuals is an important next step to explore. Further, the high endorsement rate of problems with foods types that are hypothesized to be more addictive (e.g., high fat sweets) suggests that future research is needed to understand the role of the food triggering addictive eating and foresee treatment outcome for individuals with eating disorders.

## Data availability statement

The datasets presented in this article are not readily available because of the protection law for the anonymized data of public hospital patients in Spain. Requests to access the datasets should be directed to FF-A, ffernandez@bellvitgehospital.cat.

## Ethics statement

The studies involving human participants were reviewed and approved by Clinical Research Ethics Committee of the University Hospital of Bellvitge. The patients/participants provided their written informed consent to participate in this study.

## Author contributions

LM, LC-B, SJ-M, and FF-A: conceptualization. RG: methodology, formal analysis, and data curation. LM, LC-B, and AG-P: investigation. SJ-M and FF-A: resources. LM, LC-B, AG-P, SJ-M, RG, and FF-A: writing – original draft preparation. LM, SJ-M, AG, and FF-A: writing – review and editing. LM, SJ-M, CD, AG, and FF-A: supervision. SJ-M and FF-A: funding acquisition. All authors have read and agreed to the published version of the manuscript.
