# Supplementary material for: Food addiction in Bulimia Nervosa: Analysis of body composition, psychological and problematic foods profile
Source: Front Psychiatry. 2022 Oct 20;13:1032150. doi: 10.3389/fpsyt.2022.1032150 (PMC9632855; doi:10.3389/fpsyt.2022.1032150)
Supplement: Supplementary file 1 [file Table_1.DOCX]

***Table S1 (supplementary)*** *Complete results in the SEM: test for total, direct and indirect effects*

|  |  |  | Total effects | | | | | | Direct effects | | | | | | Indirect effects | | | | | |
| --- | --- | --- | --- | --- | --- | --- | --- | --- | --- | --- | --- | --- | --- | --- | --- | --- | --- | --- | --- | --- |
|  |  |  | *Coeff* | *SE* | *z-stat* | *p* | *95% CI (coeff)* | | *Coeff* | *SE* | *z-stat* | *p* | *95% CI (coeff)* | | *Coeff* | *SE* | *z-stat* | *p* | *95% CI (coeff)* | |
| Structural | SCL-90R GSI | DERS-total | 0.010 | 0.002 | 4.08 | <.001 | 0.005 | 0.014 | 0.010 | 0.002 | 4.08 | <.001 | 0.005 | 0.014 | *** | *** | *** | *** | *** | *** |
|  |  | FA (present) | 0.379 | 0.176 | 2.16 | .031 | 0.035 | 0.724 | 0.379 | 0.176 | 2.16 | .031 | 0.035 | 0.724 | *** | *** | *** | *** | *** | *** |
|  | Fat mass | EDI-2 total | 0.061 | 0.020 | 3.00 | .003 | 0.021 | 0.101 | 0.061 | 0.020 | 3.00 | .003 | 0.021 | 0.101 | *** | *** | *** | *** | *** | *** |
|  |  | Foods | 0.061 | 0.020 | 3.00 | .003 | 0.021 | 0.101 | *** | *** | *** | *** | *** | *** | 0.061 | 0.020 | 3.00 | .003 | 0.021 | 0.101 |
|  |  | DERS-total | 0.035 | 0.016 | 2.27 | .023 | 0.005 | 0.066 | *** | *** | *** | *** | *** | *** | 0.035 | 0.016 | 2.27 | .023 | 0.005 | 0.066 |
|  |  | FA (present) | 1.586 | 0.919 | 1.73 | .085 | -0.216 | 3.388 | *** | *** | *** | *** | *** | *** | 1.586 | 0.919 | 1.73 | .085 | -0.216 | 3.388 |
|  | Foods | DERS-total | 0.096 | 0.052 | 1.86 | .063 | -0.005 | 0.197 | 0.096 | 0.052 | 1.86 | .063 | -0.005 | 0.197 | *** | *** | *** | *** | *** | *** |
| Measurement | Sweets-high-fat | Foods | 0.011 | 0.007 | 1.69 | .092 | -0.002 | 0.025 | 0.011 | 0.007 | 1.69 | .092 | -0.002 | 0.025 | *** | *** | *** | *** | *** | *** |
|  |  | DERS-total | 0.001 | 0.001 | 1.57 | .116 | 0.000 | 0.002 | *** | *** | *** | *** | *** | *** | 0.001 | 0.001 | 1.57 | .116 | 0.000 | 0.002 |
|  | Salty-high-fat | Foods | 0.011 | 0.010 | 1.16 | .245 | -0.008 | 0.030 | 0.011 | 0.010 | 1.16 | .245 | -0.008 | 0.030 |  |  |  |  |  |  |
|  |  | DERS-total | 0.001 | 0.001 | 1.14 | .254 | -0.001 | 0.003 | *** | *** | *** | *** | *** | *** | 0.001 | 0.001 | 1.14 | .254 | -0.001 | 0.003 |
|  | Starches | Foods | 0.021 | 0.012 | 1.71 | .087 | -0.003 | 0.044 | 0.021 | 0.012 | 1.71 | .087 | -0.003 | 0.044 |  |  |  |  |  |  |
|  |  | DERS-total | 0.002 | 0.001 | 1.52 | .128 | -0.001 | 0.005 | *** | *** | *** | *** | *** | *** | 0.002 | 0.001 | 1.52 | .128 | -0.001 | 0.005 |
|  | Sweets | Foods | 0.046 | 0.022 | 2.12 | .034 | 0.003 | 0.088 | 0.046 | 0.022 | 2.12 | .034 | 0.003 | 0.088 |  |  |  |  |  |  |
|  |  | DERS-total | 0.004 | 0.002 | 1.84 | .065 | 0.000 | 0.009 | *** | *** | *** | *** | *** | *** | 0.004 | 0.002 | 1.84 | .065 | 0.000 | 0.009 |
|  | Control | Foods | 0.019 | 0.015 | 1.26 | .209 | -0.011 | 0.049 | 0.019 | 0.015 | 1.26 | .209 | -0.011 | 0.049 |  |  |  |  |  |  |
|  |  | DERS-total | 0.002 | 0.002 | 1.17 | .243 | -0.001 | 0.005 | *** | *** | *** | *** | *** | *** | 0.002 | 0.002 | 1.17 | .243 | -0.001 | 0.005 |
| Structural | EDI-2 total | Foods | 1.000 | *const.* |  |  |  |  | 1.000 | *const.* |  |  |  |  | *** | *** | *** | *** | *** | *** |
|  |  | DERS-total | 0.577 | 0.166 | 3.49 | <.001 | 0.253 | 0.902 | 0.481 | 0.168 | 2.86 | .004 | 0.151 | 0.812 | 0.096 | 0.052 | 1.86 | .063 | -0.005 | 0.197 |
|  |  | FA (present) | 25.852 | 12.257 | 2.11 | .035 | 1.829 | 49.874 | 25.852 | 12.257 | 2.11 | .035 | 1.829 | 49.874 | *** | *** | *** | *** | *** | *** |

*Note.* Const.: constrained parameter. *** No path.
